# Evaluation of Prognostic Factors in Myxoid Liposarcoma Treated with Combined Neoadjuvant Radiotherapy and Surgical Excision: Systematic Review

**DOI:** 10.3390/diseases13060177

**Published:** 2025-06-06

**Authors:** Giuseppe Francesco Papalia, Giulia De Marco, Claudia Luciano, Luisana Sisca, Pasquale Farsetti, Bruno Vincenzi, Rocco Papalia

**Affiliations:** 1 Research Unit of Orthopaedic and Trauma Surgery, Department of Medicine and Surgery, Università Campus Bio-Medico di Roma, Via Alvaro del Portillo 21, 00128 Rome, Italy; g.papalia@unicampus.it (G.F.P.); giulia.demarco@unicampus.it (G.D.M.); r.papalia@policlinicocampus.it (R.P.); 2 Oncological Orthopaedics Department, IFO—IRCCS Regina Elena National Cancer Institute, Via Elio Chianesi 53, 00144 Rome, Italy; 3 Operative Research Unit of Orthopaedic and Trauma Surgery, Fondazione Policlinico Universitario Campus Bio-Medico, Via Alvaro del Portillo 200, 00128 Rome, Italy; 4 Section of Orthopaedics and Traumatology, Department of Clinical Science and Translational Medicine, University of Rome “Tor Vergata”, 00133 Rome, Italy; farsetti@uniroma2.it; 5 Department of Medical Oncology, Fondazione Policlinico Universitario Campus Bio-Medico, Via Alvaro del Portillo, 200, 00128 Rome, Italy; luisana.sisca@unicampus.it (L.S.); b.vincenzi@policlinicocampus.it (B.V.)

**Keywords:** myxoid liposarcoma, neoadjuvant radiotherapy, surgical margins, prognostic factors, systematic review

## Abstract

Purpose: Myxoid liposarcoma (MLPS) is a malignant tumor that occurs predominantly in the deep soft tissues of the extremities. Preoperative radiotherapy (RT) is used to reduce tumor volume to achieve adequate surgical margins. This systematic review aims to evaluate the impact of preoperative RT on surgical margins, local recurrence (LR) rates, metastasis development, and overall survival in patients with MLPS and associated prognostic factors. Methods: A systematic literature search was conducted by two reviewers following PRISMA guidelines on PubMed, Scopus, and the Cochrane Library on 30 November 2024. We included prospective and retrospective cohort studies published in English that evaluate surgical margin status, LR and metastasis rates, and survival outcomes in patients undergoing surgical excision of MLPS following neoadjuvant radiotherapy. Two authors extracted tumor characteristics, percentage of round cells (RCs), change in tumor volume post-RT, surgical margins, postoperative complications, LR and metastasis rates, survival rates, and related prognostic factors. Results: The twelve studies included in this review involved 1483 patients with a mean age of 44.8 years. Tumors were mostly located in the lower limbs, deeply localized, and larger than 5 cm in most cases. The average LR and metastasis rates were 5.2% and 17%, respectively. The mean 5-year and 10-year overall survival rates were 87% and 74%, respectively. Poor prognosis was associated with >5% RC components, tumors larger than 15 cm, deep localization, and inadequate surgical margins. Conclusion: The management of MLPS requires a multidisciplinary approach. Preoperative radiotherapy offers several advantages in reducing tumor volume and facilitating the achievement of adequate surgical margins, finally improving local control and long-term outcomes.

## 1. Introduction

Myxoid liposarcoma (MLPS) is a malignant tumor consisting of uniform round-to-oval cells and small lipoblasts embedded in a myxoid stroma [1]. Its pathognomonic feature is evidence of the FUS-DDIT3 gene fusion, due to the translocation t(12;16)(q13;p11), or, less commonly, EWSR1-DDIT3 [2]. Myxoid liposarcoma accounts for approximately 20–30% of all liposarcomas and is the most common subtype of liposarcoma in the pediatric population [3]. Diagnosis can be challenging in the presence of numerous round cells [4]. MLPS predominantly occurs in the deep soft tissues of the extremities, most frequently in the thigh. Following initial diagnostics with ultrasound, Magnetic Resonance Imaging (MRI) is the gold standard for diagnosing liposarcoma. Preoperative radiotherapy is used to reduce tumor volume and facilitate adequate surgical margin achievement [5]. The integration of surgery and radiation therapy (RT) is highly effective in reducing the local recurrence (LR) rate of MLPS, given its elevated sensitivity to both RT and chemotherapy [6]. MLPS exhibits greater radiosensitivity compared to other soft tissue sarcomas (STSs) [7]. Therefore, this combined treatment strategy offers better outcomes in terms of local control and survival compared to the majority of other STSs. Additionally, the synergistic activity of RT and trabectedin in MLPS has been analyzed [8]: trabectedin enhances radiosensitivity, inducing S-phase block and G2/M arrest [9]. The current cornerstone of treatment is a combination of preoperative radiotherapy and surgical resection, which has shown high rates of local tumor control and improved oncological outcomes [10]. The adequacy of surgical margins for MLPS in irradiated extremities is unclear [11].

Prognosis in patients with MLPS is influenced by several factors such as age, tumor size, the depth of the tumor, the status of surgical margins, and morphological characteristics like tumor grade, percentage of round cell component, presence of necrosis, mitotic activity, proliferation index, and P53 overexpression [12,13,14]. Local recurrence occurs in 12–25% of cases, while distant metastases can develop in 30–60% of cases—even several years after the initial diagnosis—with a preference for soft tissues and bones (particularly the spine) [15]. Given the propensity of MLPS to metastasize preferentially to non-pulmonary sites, whole-body MRI scans are increasingly performed for newly diagnosed MLPS patients and for staging purposes [16]. This systematic review aims to evaluate the impact of preoperative radiotherapy on surgical margins, local recurrence rates, metastasis development, and overall survival in patients with myxoid liposarcoma and associated prognostic factors.

## 2. Materials and Methods

### 2.1. Search Strategy

A comprehensive literature search was performed on 30 November 2024 by two reviewers (G.F.P. and C.L.) on PubMed, Scopus, and Cochrane Library using the search strings (“liposarcoma, myxoid”[MeSH Terms] OR (“liposarcoma”[All Fields] AND “myxoid”[All Fields]) OR “myxoid liposarcoma”[All Fields] OR (“myxoid”[All Fields] AND “liposarcoma”[All Fields])) AND (“radiotherapy”[MeSH Subheading] OR “radiotherapy”[All Fields] OR (“radiation”[All Fields] AND “therapy”[All Fields]) OR “radiation therapy”[All Fields] OR “radiotherapy”[MeSH Terms]).

Duplicates were removed, manuscripts were screened by title and abstract, and then potentially eligible articles were read in full to select studies for inclusion in this review.

### 2.2. Inclusion Criteria

We included prospective and retrospective cohort studies published in English that evaluated surgical margin status, local recurrence and metastasis rates, and survival outcomes in patients undergoing surgical excision of myxoid liposarcoma of the extremity following neoadjuvant radiotherapy.

Studies were excluded if they did not differentiate between MLPS and other histological subtypes of STS, did not clearly delineate preoperative RT, focused on metastatic MLPS, did not report oncological outcome data, or were limited to histological analyses.

### 2.3. Data Collection

Two authors (G.D.M. and C.L.) extracted the following from the included studies: the type of study, number/mean age/gender of patients, site/size/grade/depth of the tumor, percentage of round cells (RCs), radiation dose, change in tumor volume post-RT, other neo-adjuvant or adjuvant treatments (and chemotherapy protocols), surgical margins, postoperative complications, follow-up, local recurrence and metastasis rates, survival rates, and related prognostic factors.

## 3. Results

### 3.1. Search Results

**Figure 1:**
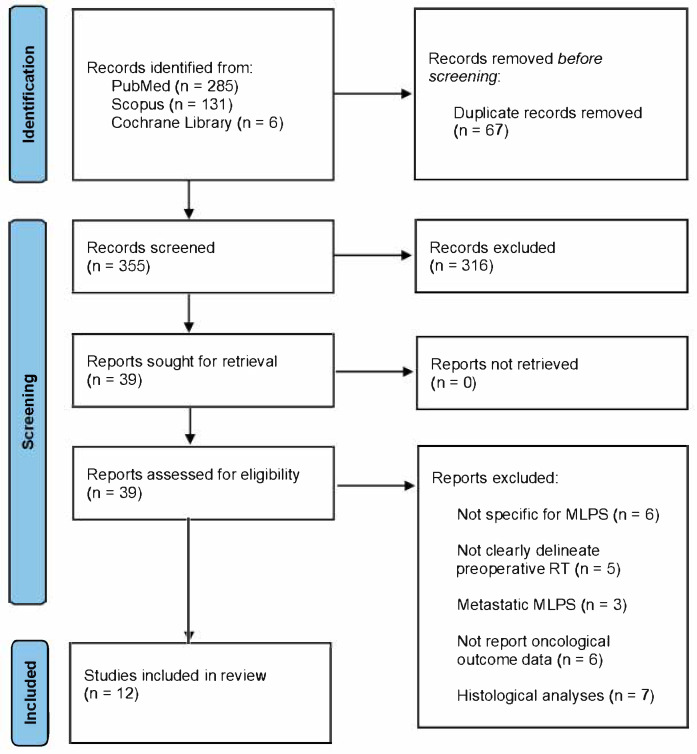
PRISMA 2020 flow diagram.

The literary search found 422 papers. After the removal of 67 duplicates, 355 manuscripts were screened by title and abstract. Then, 39 studies were evaluated in full and 26 were excluded due to not being specific to MLPS (n = 6), not clearly delineating preoperative RT (n = 5), focusing on metastatic MLPS (n = 3), not reporting oncological outcome data (n = 6), or being histological analyses (n = 7). Finally, 12 articles were included in this review: 10 retrospective and 2 prospective studies (Figure 1).

### 3.2. Demographic Data

This study included 1483 patients (54% male and 46% female). The mean age of the patients was 44.8 years (range 36–56.3). The minimum and the maximum follow-up were 25 and 109.2 months, with a mean of 68 months (Table 1).

### 3.3. Tumor Characteristics

The most frequently involved location was the lower limb, and tumors were in most cases deeply localized. The tumor size was greater than 5 cm in most cases. The median tumor size was documented for 827 patients and ranged from 9.9 to 15 cm, and the mean size was 12 cm (Table 2).

### 3.4. Radiotherapy and Chemotherapy Treatment

In all included studies, preoperative and—in some cases—postoperative RT was administered. The mean percentage of patients who received preoperative RT followed by surgery was 70% (mean of 47 Gy). Moreover, an average of 46% of patients received adjuvant RT (mean of 52.4 Gy). In two studies RT was delivered preoperatively with a postoperative boost [10,22]. A mean of 37% of patients received chemotherapy in combination with RT, and the most commonly used protocol was Doxorubicin and Ifosfamide.

Margins were assessed both macroscopically and microscopically in all dimensions and were determined to be R0, R1, and R2 with an average rate of 74.3%, 19.1%, and 4.6%, respectively.

### 3.5. Local Recurrence and Metastasis

The average local recurrence and metastasis rates were 5.2% and 17%, respectively. The mean time to local recurrence and metastases was 27 months and 29.4 months, respectively.

The metastasis-free survival was reported in three studies, and the mean 5-year metastasis-free survival rate was 84%. The local recurrence-free survival was reported in eight studies, and the mean 5-year and 10-year local recurrence-free survival rates were 95% and 91%, respectively.

The most common complication was wound complication (mean 18%, range 9.5–25%) (Table 3).

### 3.6. Survival Rates

The overall survival (OS) rate was evaluated in five studies: the mean 5-year and 10-year OS rates were 87% and 74%, respectively.

The disease-free survival rate was evaluated in four studies: the mean 5-year and 10-year disease-free survival rates were 73% and 68%, respectively.

The disease-specific survival (DSS) rate was evaluated in six studies: the mean 5-year and 10-year DSS rates were 88% and 82%, respectively (Table 3).

### 3.7. Prognostic Factors

Several prognostic factors were evaluated in patients with MLPS treated using combined neoadjuvant radiotherapy and surgical excision (Table 4).

#### 3.7.1. Round Cells

The most significant factor affecting prognosis was the occurrence of round cell components (RC >5% carried the worst prognosis). In the reviewed studies the mean 5-year metastasis-free survival rate was 84% (*p* = 0.003) [23] and 27% (*p* = 0.052) [25] in patients with >5% RC.

In patients with >25% RC the mean 5-year metastasis-free survival rate was 69% (*p* = 0.025) [23].

In the >5% RC group the rate of OS decreased dramatically from 91.7% at 2 years to 50% at 10 years, and it was found to be an independent risk factor for decreased OS [17].

The round cell component remained a highly significant prognostic factor.

#### 3.7.2. Tumor Grade

Moreover, the tumor grade was found to be an independent prognostic factor for the development of metastases: 5-year metastasis-free survival rates were 90%, 87%, and 67% for grade 1, grade 2, and grade 3 (*p* = 0.03), respectively [23]. The 10-year OS rate was 86.3% in low-grade patients and 46.2% in high-grade patients (*p* = 0.022) [17].

#### 3.7.3. Tumor Depth

The mean 5-year metastasis-free survival rate was 78% for deep tumors and 96% for superficial tumors [23].

#### 3.7.4. Tumor Size

Tumor size had a significant impact on the survival rate.

Tumor size >15 cm was significantly associated with decreased metastasis-free survival: the average metastasis rate was 83% in patients with >15 cm tumor size (*p* = 0.006) [25].

#### 3.7.5. Marginal Status

Obtaining a good surgical margin is the most important factor in maintaining local control.

The resection margins were determined as R0, R1, and R2, with the 5-year LR-free survival rates of 95%, 83%, and 43%, respectively [23], and with 10-year OS rates of 94.4%, 65.5%, and 33%, respectively (*p* = 0.024) [25].

## 4. Discussion

Myxoid liposarcoma is a rare and clinically distinctive subtype of liposarcoma, primarily affecting soft tissues, with a notable predilection for the lower limbs [28]. Although MLPS is considered less aggressive than other soft tissue sarcomas, it still carries a significant risk for distant metastases, and is also associated with high rates of local recurrence. Myxoid liposarcoma also differs from conventional subtypes due to its propensity for extrapulmonary metastases [6]. This metastatic potential remains a critical factor in prognosis and long-term patient management [14]. Moreover, the tumor’s infiltrative growth pattern, combined with its tendency to form large masses, presents significant challenges for surgical resection, making complete excision difficult in many cases [29].

Additionally, the tumor’s high vascular density is a critical histological feature that directly correlates with its biological aggressiveness and its ability to metastasize. The increased blood supply also enhances its sensitivity to radiotherapy, which can potentially exploit this feature to improve treatment outcomes [30]. Furthermore, the tumor vascular profile not only influences its radiosensitivity but also impacts its overall behavior, including its tendency to metastasize to distant organs, further complicating treatment.

The management of MLPS is inherently multidisciplinary, combining surgery, radiotherapy, and, in selected cases, chemotherapy. The cornerstone of treatment remains surgical resection with clear margins, although the infiltrative nature of the tumor often complicates complete excision, particularly when the tumor is large or located in deep tissues [31]. Surgical planning must therefore account for both the tumor’s size and its anatomical location, aiming for negative surgical margins to optimize the likelihood of a complete resection and minimize the risk of local recurrence [32]. Given that MLPS exhibits significant radiosensitivity, radiotherapy (RT) is a critical adjunct to surgery, both in the preoperative and postoperative settings. Preoperative RT is particularly advantageous in reducing tumor volume, thus enhancing surgical resection by facilitating easier dissection and improving the likelihood of obtaining negative margins. Preoperative RT not only reduces tumor size but also improves the definition of surgical margins, which can help reduce the risk of local recurrence post-surgery [33]. Additionally, preoperative radiotherapy is associated with better overall survival outcomes by reducing tumor growth and the risk of metastasis, thereby improving patient prognosis [34]. In our review, the preoperative radiotherapy ranges from 28 to 54 Gy, with a mean dose of 47 Gy, with a total dose typically between 50 and 60 Gy; however, dose de-escalation is possible in myxoid liposarcomas [33]. The Phase II DOREMY trial, which applied a preoperative dose of 36 Gy in 2 Gy fractions over 3–4 weeks, achieved excellent local control rates of 100% after a median follow-up of 25 months [27].

The postoperative radiotherapy approach remains an important modality, particularly when complete excision is not feasible or when surgical margins are marginal. Moreover, postoperative RT is typically administered at a dose of around 60 Gy, particularly when tumors are large (>5 cm) or located deeply within the tissue. This approach helps mitigate the risk of local recurrence, especially when negative margins cannot be achieved during surgery [35]. However, the literature presents conflicting data regarding radiotherapy techniques. While some studies suggest potential advantages in terms of local control or toxicity profiles with specific techniques, other evidence indicates that outcomes between different approaches may be largely comparable [26].

Chemotherapy plays a relatively limited role in the management of MLPS. It is typically reserved for advanced or metastatic disease or in cases of tumor recurrence. The most used chemotherapy regimen in this context includes doxorubicin and ifosfamide, two well-established agents for soft tissue sarcomas. Approximately 34% of patients in studies evaluating combined treatment approaches received chemotherapy alongside radiotherapy [36]. However, chemotherapy is generally not the first-line treatment, as surgery and radiotherapy remain the primary modalities for local control. Its role is more pronounced in cases of advanced, metastatic, or recurrent disease, where it may help manage disease spread and control symptoms.

The aim of this systematic review was to evaluate the impact of preoperative RT on margin status and long-term outcomes in patients with myxoid liposarcoma and associated prognostic factors. Haniball et al. [37] in their study investigated prognostic factors in patients undergoing surgical excision and postoperative radiotherapy for myxoid/round-cell liposarcoma. They report that the RC component is the most significant predictor of behavior of the tumor for MLPS; patients with >5% RC component were at higher risk of LR, metastasis, and tumor-related death, and should have been considered for adjuvant radiotherapy and possibly chemotherapy. Additionally, pure myxoid liposarcoma with no round cell component had a very good prognosis in their study, with no tumor-related death reported after the first 18 months.

Moreover, Lansu et al. [38] in their population-based study included both patients with primary localized and metastatic MLPS diagnosed between 1989 and 2016. They revealed a higher probability of having a RC tumor for older patients and an increase of approximately 2% per additional year of age; independent prognostic factors included age, tumor size, and location. They reported an increase in the use of preoperative radiotherapy with significant improvements in overall survival over time in metastatic cases.

Among the studies included in our review, Buyukceran et al. [17] observed that high tumor grade, >5% RC, R2 resection margins, and LR had a statistically significant negative effect on OS (*p* < 0,05). Round cell >5% (*p* = 0.003), round cell >25% (*p* = 0.025), tumor grade (*p* = 0.03), and tumor diameter >10 cm (*p* = 0.01) were all found to be independent prognostic factors for the development of metastases. Tumor grade also had a significant impact on survival (*p* = 0.04). On multivariate analysis, age at diagnosis (>45 years; *p* = 0.047), tumor diameter (>10 cm; *p* = 0.024), and round cell percentage (>5%; *p* = 0.02) had a significant impact on the survival rate [23]. Gronchi et al. in their phase 1 study used a dose of RT slightly lower (45 Gy) than the one conventionally used in sarcomas in the preoperative setting (50 Gy) [18]. Age > 45, male gender, and locally recurrent disease were statistically significant prognosticators of a poor outcome for DSS [20]. Radiotherapy prevented local relapse (*p* < 0.001). Both use of radiotherapy and the microscopic margin status were strong independent predictors for local recurrence (*p* < 0.001) [23].

This study has several limitations. Although the majority of patients received preoperative radiotherapy followed by surgical resection, the included studies did not stratify oncological outcomes based on the use of concomitant chemotherapy or postoperative radiotherapy. Therefore, it was not possible to assess the potential confounding effects of these additional treatments. Another limitation of this review is the heterogeneity in radiotherapy and chemotherapy protocols among included studies. Indeed, although the most used preoperative dose was between 45 and 50 Gy, only two studies [25,27] adopted hypo-fractionated regimens or dose de-escalation strategies.

## 5. Conclusions

The management of myxoid liposarcoma requires a multidisciplinary approach. Preoperative radiotherapy offers several advantages in reducing tumor volume and facilitating the achievement of adequate surgical margins, improving local control and long-term outcomes. Poor prognosis was associated with >5% round cell components, tumors larger than 15 cm, deep localization, and inadequate surgical margins.

## Figures and Tables

**Table 1:** Demographic data and tumor characteristics.

Author (Year)	Type of Study	LOE	N	Age	Sex	Site	Tumor Size	Grade	Tumor Depth	% Round Cell
Buyukceran et al. (2022) [17]	ROS	III	43	56.3	32.5% F, 67.5% M	Upper limb (23.2%), Lower limb (76.7%)	0–5 cm (18.6%)	Low (37.2%), High (62.7%)	N.R.	0 (18.6%), 0–5 (53.5%)
Chung et al. (2009) [10]	ROS	III	88	48	41% F, 59% M	\	\	Grade 2 (93.2%), Grade 3 (6.8%)	Superficial (15%), Deep (85%)	\
Gronchi et al. (2019) [18]	POS	II	14	36	50% F, 50% M	Tigh (71%), Leg (21%), Buttock (7%)	>10 cm (100%)	\	Deep (100%)	\
Guadagnolo et al. (2008) [19]	ROS	III	127	41	43% F, 57% M	Head and neck (2%), Superficial trunk (10%), Deep trunk (2%), Upper limb (3%), Lower limb (83%)	≤5 (15%), >5 (85%), ≤10 (57%), >10 (43%)	\	\	\
Hoffman et al. (2013) [20]	ROS	III	207	41	42.5% F, 57.5% M	Extremities (74%), Other (26%)	\	\	Deep (76%), Superficial (24%)	\
Lee et al. (2022) [21]	ROS	III	24	39.9	75% F, 25% M	Thigh (91.7%), Calf (8.3%)	\	\	\	Present (16.7%), Not present (75%), Unknown (8.3%)
Houdek et al. (2023) [22]	ROS	III	62	47 ± 14	45% F, 55% M	Lower extremity (97%), Upper extremity (3%)	\	Low (81%), High (19%)	Deep (100%)	0 (81%), 0–5 (16.1%), ≥5 (2.9%)
Moreau et al. (2012) [23]	ROS	III	418	45	41% F, 59% M	Lower limb (90%), Upper limb (7%), Chest wall (1%), Paraspinal (1%)	<5 cm (13.15%), 5–10 cm (33.25%), >10 cm (53.6%)	Grade 1 (33%), Grade 2 (49%), Grade 3 (14%), Unknown (4%)	Deep (81%), Superficial (18%)	Pure myxoid (74%), Round cell (26%)
Perera et al. (2023) [24]	ROS	III	198	46	34% F, 66% M	Lower extremity (97%), Upper extremity (3%)	\	Grade 1 (16%), Grade 2 (62%), Grade 3 (19%)	\	<5% (73%), ≥5% (15%)
Salduz et al. (2017) [25]	ROS	III	23	43	30.4% F, 69.6% M	Lower extremities (95.6%), Upper extremities (4.3%)	\	\	\	Round cell (21.7%)
Masunaga et al. (2023) [26]	ROS	III	200	<40 years (25%), 40–65 years (59%), >65 years (16%)	38.5% F, 61.5% M	Lower limb (78%), Upper limb (5.5%), Trunk (16.5%)	<5 cm (4.5%), 5–10 cm (38%), 10–15 cm (30.5%), ≥15 cm (27%)	High (67%), Low (33%)	Deep (94%), Superficial (6%)	\
Lansu et al. (2021) [27]	POS		79	45	44% F, 56% M	Lower extremity proximal (78%), Lower extremity distal (13%), Upper extremity (3%), Trunk (6%)	\	\	\	<5% (77%), >5% (19%), Unknown (4%)

LOE: level of evidence; ROS: retrospective observational study; POS: prospective observational study; M: male; F: female; \: not reported.

**Table 2:** Treatment regimen and surgical margins.

Author (Year)	Tumor Dimension Pre- and Post-RT	RT Gy	RT	Chemotherapy	CT Protocols	Margins (%)
Buyukceran et al. (2022) [17]	\	45–50 Gy pre	Neo (32.5%)	9.3%	doxorubicin + ifosfamide	R0 (41.9%) R1 (39.5%) R2 (18.6%)
		60 Gy post	Adj (67.4%)			
Chung et al. (2009) [10]	\	50 Gy pre	Neo (47%),	\	\	R0 (81%) R1 (19%)
		60–66 Gy post	Adj (43%), Neo + boost postop (10%)			
Gronchi et al. (2019) [18]	Size pre-RT: 12.5 cm	45 Gy pre	Neo (100%)	100%	trabectedin (median dose 2.6 mg per cycles)	R0 (58%) R1 (42%)
		16 Gy post (if R1)				
Guadagnolo et al. (2008) [19]	\	50 Gy pre	Neo (54%)	28%	doxorubicin based + cyclophosphamide or vincristine or dacarbazine or ifosfamide	R0 (72%) R1-R2 (9%) Unknown (20%)
		60 Gy post	Adj (46%)			
Hoffman et al. (2013) [20]	Size pre-RT: 10 ± 6.7 cm	\	Neo 57%, Adj 42%, No surgery 1%	Neo (77%), Adj (17%)	doxorubicin + ifosfamide	R0 (83%), R1 (13%), R2 (4%)
Lee et al. (2022) [21]	Size pre-RT: 12.4 cm; Volume pre-RT: 298.9 cm^3^; Size post-RT: 8.7 cm; Volume post-RT: 106.9 cm^3^	50 Gy pre		Neo (16.7%)	doxorubicin + ifosfamide	\
Houdek et al. (2023) [22]	Size pre-RT 15 ± 6 cm; Volume pre-RT: 629 ± 631 cm^3^; Size post-RT: 12 ± 5 cm; Volume post-RT: 304 ± 387 cm^3^	50.4 Gy pre	Neo (55%), Neo + Boost intraop (34%), Neo + boost postop (11%)	21%	doxorubicin + ifosfamide + mitomycin C and cisplatin	R0 (87%), R1 (3.2%), R2 (9.7%)
Moreau et al. (2012) [23]	Size pre-RT 10 cm; Volume pre-RT 325 cm^3^	50 Gy pre	Neo (40.7%)	6%	\	R0 (73.9%) R1 (22%) R2 (3.3%)
		65 Gy post	Adj (28.2%)			
		66 Gy	Neo + Adj (5%)			
Perera et al. (2023) [24]	Volume pre-RT: 240.6 cm^3^; post-RT volume change: 60.1%	50 Gy pre	Neo (100%)			Micro positive (11%) <1 mm (15%) ≥1 mm (72%)
Salduz et al. (2017) [25]	Size pre-RT: 13.7 cm	28 Gy pre	Neo (100%)	Neo (34.8%)	adriamycin + ifosfamide (2 Cycles)	Wide (82.6%) Marginal (17.4%)
Masunaga et al. (2023) [26]	\	30–56 Gy pre	Neo (50%)	33.5%: Neo (31.4%), Adj (40.3%), Neo + Adj (28.3%)	anthracycline + ifosfamide; anthracycline	R0 (71.5%) R1 (24.5%) R2 (4%)
		45–70 Gy post	Adj (50%)			
Lansu et al. (2021) [27]	Size pre-RT: 9.9 cm	36 Gy pre	Neo (99%)	\	\	R0 (94%) R1 (6%)
		30 Gy post	Neo + Adj (1%)			

**Table 3:** Local recurrence, metastasis, and survival rates.

Author (Year)	Complications (%)	Follow-Up (m)	% LR	Time to LR (m)	LR-Free Survival	% METS	Time to METS (m)	METS-Free Survival	Disease-Free Survival	DSS	Overall Survival
Buyukceran et al. (2022) [17]	Wound necrosis (14%)	106.8	27.9	22.8	\	9.3	\	\	\	\	2 Y (93%) 5 Y (81.2%) 10 Y (72.7%)
Chung et al. (2009) [10]	\	86	2.27	\	5 Y (97.7%)	13.6	\	5 Y (89.1%)	\	\	5 Y (93.9%)
Gronchi et al. (2019) [18]	Sepsis (1%)	26	7.14	\	3 Y (92%)	7.14	15	\	3 Y (86%)	\	3 Y (93%)
Guadagnolo et al. (2008) [19]	Edema (4%), Cellulitis (1%), Fibrosis (1.57%), Stress fracture (2.4%)	109.2	3	14	\	21	27	5 Y (85%) 10 Y (76%)	5 Y (81%) 10 Y (73%) 15 Y (71%)	5 Y (89%) 10 Y (83%) 15 Y (78%)	5 Y (87%) 10 Y (79%) 15 Y (71%)
Hoffman et al. (2013) [20]	\	68	7.4	31	\	13	34	\	\	1 Y (99%) 5 Y (93%) 10 Y (87%)	\
Lee et al. (2022) [21]	Wound complications (25%)	\	\	\	\	\	\	\	\	\	\
Houdek et al. (2023) [22]	Wound healing (21%), Radiation-associated fractures (13%), Deep infection (8%)	96	6	35	2 Y (98%) 5 Y (93%) 10 Y (90%)	29	20	\	2 Y (81%) 5 Y (68%) 10 Y (66%)	2 Y (94%) 5 Y (84%) 10 Y (76%)	\
Moreau et al. (2012) [23]	\	62.4	7	33	10 Y (93%)	20	26.4	\	5 Y (77%)	5 Y (85%)	\
Perera et al. (2023) [24]	18%	60.7	1	17	5 Y (98.9%)	24.24	\	5 Y (77.2%)	\	\	5 Y (94.2%)
Salduz et al. (2017) [25]	\	55.1	4	52	5 Y (91%) 10 Y (91%)	26	51	\	5 Y (66%) 10 Y (66%)	\	5 Y (78.1%) 10 Y (71%)
Masunaga et al. (2023) [26]	Wound complications (9.5%)	40.5	6.5 (RT Neo) 9 (RT Adj)	21 (RT Neo) 14 (RT Adj)	5 Y (94.9%)	15.5	\	\	\	5 Y (88.1%)	\
Lansu et al. (2021) [27]	Wound complications (22%)	25	0	\	\	2.5	\	\	1 Y (97%) 3 Y (93%)	1 Y (99%) 3 Y (96%)	1 Y (99%) 3 Y (95%)

LR: local recurrence; METS: metastasis; \: not reported; m: months; Y: year; DSS: disease-specific survival.

**Table 4:** Prognostic factors.

Prognostic Factors	Authors			
	Buyukceran et al. (2022) [17]	Moreau et al. (2012) [23]	Perera et al. (2023) [24]	Salduz et al. (2017) [25]
LRFS and depth	\	\	\	\
LRFS and size	\	\	\	\
LRFS and histology	\	\	\	\
LRFS and margin	\	5 Y: R0 (95%), R1 (83%), R2 (43%)	5 Y: microscopically positive (100%), R1 < 1 mm (96.4%), R1 > 1 mm (99.3%)	\
MT and size	\	\	\	>15 cm (83%) [*p* = 0.006]
MFS and margins	\	\	5 Y: microscopically positive (67,5%), R1 < 1 mm (74,4%), R1 > 1 mm (79%)	Wide (higher MFS) [*p* = 0.023]
MFS and grade	\	5 Y: grade 1 (90%), grade 2 (87%), grade 3 (67%)	\	\
MFS and depth	\	5 Y: deep (78%), superficial (96%)	\	\
MFS and histology	\	5 Y: >5% RC (84%), >25% (69%)	\	5 Y: RC (27%) [*p* = 0.052]
DSS and histology	\		\	\
OS and %RC	2 Y: >5% (91.7%) 10 Y: >5% (50%)	\	\	5 Y: RC (53%)
OS and grade	10 Y: low-grade (86.3%), high-grade (46.2%)	\	\	\
OS and surgical margin	10 Y: R0 (94.4%), R1 (62.5%), R2 (33%)	\	\	\

LRFS: local recurrence-free survival: MFS: metastasis-free survival; DSS: disease-specific survival; OS: overall survival; RC: round cell. Discussion.

## Abbreviations

The following abbreviations are used in this manuscript:

MLPS	Myxoid Liposarcoma
RT	Radiotherapy
LR	Local Recurrence
RCs	Round Cells

## References

[1] Muratori F., Bettini L., Frenos F., Mondanelli N., Greto D., Livi L., Franchi A., Roselli G., Scorianz M., Capanna R. (2018). Myxoid Liposarcoma: Prognostic Factors and Metastatic Pattern in a Series of 148 Patients Treated at a Single Institution. Int. J. Surg. Oncol..

[2] Hou X., Shi W., Luo W., Luo Y., Huang X., Li J., Ji N., Chen Q. (2024). FUS::DDIT3 Fusion Protein in the Development of Myxoid Liposarcoma and Possible Implications for Therapy. Biomolecules.

[3] Lee A.T.J., Thway K., Huang P.H., Jones R.L. (2018). Clinical and Molecular Spectrum of Liposarcoma. J. Clin. Oncol..

[4] Lemeur M., Mattei J.-C., Souteyrand P., Chagnaud C., Curvale G., Rochwerger A. (2015). Prognostic Factors for the Recurrence of Myxoid Liposarcoma: 20 Cases with up to 8 Years Follow-Up. Orthop. Traumatol. Surg. Res..

[5] Le Grange F., Cassoni A.M., Seddon B.M. (2014). Tumour Volume Changes Following Pre-Operative Radiotherapy in Borderline Resectable Limb and Trunk Soft Tissue Sarcoma. Eur. J. Surg. Oncol. (EJSO).

[6] Qu G., Zhang C., Tian Z., Yao W. (2024). Diagnosis and Treatment of Myxoid Liposarcoma. Curr. Treat. Options Oncol..

[7] Pitson G., Robinson P., Wilke D., Kandel R.A., White L., Griffin A.M., Bell R.S., Catton C.N., Wunder J.S., O’Sullivan B. (2004). Radiation Response: An Additional Unique Signature of Myxoid Liposarcoma. Int. J. Radiat. Oncol. Biol. Phys..

[8] Simoens C., Korst A.E.C., De Pooter C.M.J., Lambrechts H.A.J., Pattyn G.G.O., Faircloth G.T., Lardon F., Vermorken J.B. (2003). In Vitro Interaction between Ecteinascidin 743 (ET-743) and Radiation, in Relation to Its Cell Cycle Effects. Br. J. Cancer.

[9] Romero J., Zapata I., Córdoba S., Jimeno J.M., López-Martín J.A., Tercero J.C., La Torre A.D., Vargas J.A., Molerón R., Sánchez-Prieto R. (2008). In Vitro Radiosensitisation by Trabectedin in Human Cancer Cell Lines. Eur. J. Cancer.

[10] Chung P.W.M., Deheshi B.M., Ferguson P.C., Wunder J.S., Griffin A.M., Catton C.N., Bell R.S., White L.M., Kandel R.A., O’Sullivan B. (2009). Radiosensitivity Translates into Excellent Local Control in Extremity Myxoid Liposarcoma: A Comparison with Other Soft Tissue Sarcomas. Cancer.

[11] Alaggio R., Coffin C.M., Weiss S.W., Bridge J.A., Issakov J., Oliveira A.M., Folpe A.L. (2009). Liposarcomas in Young Patients: A Study of 82 Cases Occurring in Patients Younger Than 22 Years of Age. Am. J. Surg. Pathol..

[12] Tuzzato G., Laranga R., Ostetto F., Bubbico E., Vara G., Bianchi G. (2022). Primary High-Grade Myxoid Liposarcoma of the Extremities: Prognostic Factors and Metastatic Pattern. Cancers.

[13] Lebas A., Le Fèvre C., Waissi W., Chambrelant I., Brinkert D., Noël G. (2023). Prognostic Factors in Extremity Soft Tissue Sarcomas Treated with Radiotherapy: Systematic Review of the Literature. Cancers.

[14] Shinoda Y., Kobayashi E., Kobayashi H., Mori T., Asano N., Nakayama R., Morioka H., Iwata S., Yonemoto T., Ishii T. (2020). Prognostic Factors of Metastatic Myxoid Liposarcoma. BMC Cancer.

[15] Dürr H.R., Rauh J., Baur-Melnyk A., Knösel T., Lindner L., Roeder F., Jansson V., Klein A. (2018). Myxoid Liposarcoma: Local Relapse and Metastatic Pattern in 43 Patients. BMC Cancer.

[16] Stevenson J.D., Watson J.J., Cool P., Cribb G.L., Jenkins J.P.R., Leahy M., Gregory J.J. (2016). Whole-Body Magnetic Resonance Imaging in Myxoid Liposarcoma: A Useful Adjunct for the Detection of Extra-Pulmonary Metastatic Disease. Eur. J. Surg. Oncol. (EJSO).

[17] Büyükceran İ., Erdoğan F., Karadeniz S., Sina Coşkun H., Dabak N. (2022). Evaluation of Prognostic Factors and Oncological Outcomes in Patients with Limb-Localized Myxoid Liposarcoma. Jt. Dis. Relat. Surg..

[18] Gronchi A., Hindi N., Cruz J., Blay J.-Y., Lopez-Pousa A., Italiano A., Alvarez R., Gutierrez A., Rincón I., Sangalli C. (2019). Trabectedin and RAdiotherapy in Soft Tissue Sarcoma (TRASTS): Results of a Phase I Study in Myxoid Liposarcoma from Spanish (GEIS), Italian (ISG), French (FSG) Sarcoma Groups. EClinicalMedicine.

[19] Guadagnolo B.A., Zagars G.K., Ballo M.T., Patel S.R., Lewis V.O., Benjamin R.S., Pollock R.E. (2008). Excellent Local Control Rates and Distinctive Patterns of Failure in Myxoid Liposarcoma Treated With Conservation Surgery and Radiotherapy. Int. J. Radiat. Oncol. Biol. Phys..

[20] Hoffman A., Ghadimi M.P.H., Demicco E.G., Creighton C.J., Torres K., Colombo C., Peng T., Lusby K., Ingram D., Hornick J.L. (2013). Localized and Metastatic Myxoid/Round Cell Liposarcoma: Clinical and Molecular Observations. Cancer.

[21] Lee L.H., Tepper S., Owen G., Wang D., Lopez-Hisijos N., Colman M.W., Gitelis S., Blank A.T. (2022). Radiotherapy, Volume Reduction, and Short-Term Surgical Outcomes in the Treatment of Large Myxoid Liposarcomas. Radiat. Oncol. J.

[22] Houdek M.T., Mallett K.E., Heidenreich M.J., Ahmed S.K., Wenger D.E., Smith J.H., Siontis B.L., Robinson S.I., Folpe A.L., Petersen I.A. (2023). Lack of Radiosensitivity Predicts Poor Disease Specific Survival in Myxoid Liposarcoma. J. Surg. Oncol..

[23] Moreau L.-C., Turcotte R., Ferguson P., Wunder J., Clarkson P., Masri B., Isler M., Dion N., Werier J., Ghert M. (2012). Myxoid\Round Cell Liposarcoma (MRCLS) Revisited: An Analysis of 418 Primarily Managed Cases. Ann. Surg. Oncol..

[24] Perera J.R., AlFaraidy M., Ibe I., Aoude A., Acem I., Van De Sande M.A.J., Dessureault M., Turcotte R.E., Mottard S., Basile G. (2023). Intermuscular Extremity Myxoid Liposarcoma Can Be Managed by Marginal Resection Following Neoadjuvant Radiotherapy. Eur. J. Surg. Oncol..

[25] Salduz A., Alpan B., Valiyev N., Özmen E., İribaş A., Ağaoğlu F., Bayram A., Bilgiç B., Özger H. (2017). Neoadjuvant Radiotherapy for Myxoid Liposarcomas: Oncologic Outcomes and Histopathologic Correlations. Acta Orthop. Traumatol. Turc..

[26] Masunaga T., Tsukamoto S., Honoki K., Fujii H., Kido A., Akahane M., Tanaka Y., Mavrogenis A.F., Errani C., Kawai A. (2023). Comparison of Pre-Operative and Post-Operative Radiotherapy in Patients with Localized Myxoid Liposarcoma. Jpn. J. Clin. Oncol..

[27] Lansu J., Bovée J.V.M.G., Braam P., Van Boven H., Flucke U., Bonenkamp J.J., Miah A.B., Zaidi S.H., Thway K., Bruland Ø.S. (2021). Dose Reduction of Preoperative Radiotherapy in Myxoid Liposarcoma: A Nonrandomized Controlled Trial. JAMA Oncol.

[28] Mujtaba B., Wang F., Taher A., Aslam R., Madewell J.E., Nassar S. (2021). Myxoid Liposarcoma With Skeletal Metastases: Pathophysiology and Imaging Characteristics. Curr. Probl. Diagn. Radiol..

[29] Morán L.M., Vega J., Gómez-León N., Royuela A. (2022). Myxomas and Myxoid Liposarcomas of the Extremities: Our Preliminary Findings in Conventional, Perfusion, and Diffusion Magnetic Resonance. Acta Radiol. Open.

[30] Mentzel T., Brown L.F., Dvorak H.F., Kuhnen C., Stiller K.J., Katenkamp D., Fletcher C.D.M. (2001). The Association between Tumour Progression and Vascularity in Myxofibrosarcoma and Myxoid/Round Cell Liposarcoma. Virchows Arch..

[31] Tfayli Y., Baydoun A., Naja A., Saghieh S. (2021). Management of Myxoid Liposarcoma of the Extremity (Review). Oncol. Lett..

[32] Sambri A., Caldari E., Fiore M., Zucchini R., Giannini C., Pirini M.G., Spinnato P., Cappelli A., Donati D.M., De Paolis M. (2021). Margin Assessment in Soft Tissue Sarcomas: Review of the Literature. Cancers.

[33] Roohani S., Wiltink L.M., Kaul D., Spałek M.J., Haas R.L. (2024). Update on Dosing and Fractionation for Neoadjuvant Radiotherapy for Localized Soft Tissue Sarcoma. Curr. Treat. Options Oncol..

[34] Lansu J., Braam P.M., Van Werkhoven E., Scholten A.N., Schrage Y., Van Houdt W.J., Van Langevelde K., Haas R.L. (2021). A Moderate Dose of Preoperative Radiotherapy May Improve Resectability in Myxoid Liposarcoma. Eur. J. Surg. Oncol..

[35] Roeder F. (2020). Radiation Therapy in Adult Soft Tissue Sarcoma—Current Knowledge and Future Directions: A Review and Expert Opinion. Cancers.

[36] Chowdhry V., Goldberg S., DeLaney T.F., Cote G.M., Chebib I., Kim J., Lozano-Calderón S.A., De Amorim Bernstein K. (2018). Myxoid Liposarcoma: Treatment Outcomes from Chemotherapy and Radiation Therapy. Sarcoma.

[37] Haniball J., Sumathi V.P., Kindblom L.-G., Abudu A., Carter S.R., Tillman R.M., Jeys L., Spooner D., Peake D., Grimer R.J. (2011). Prognostic Factors and Metastatic Patterns in Primary Myxoid/Round-Cell Liposarcoma. Sarcoma.

[38] Lansu J., Van Houdt W.J., Schaapveld M., Walraven I., Van De Sande M.A.J., Ho V.K.Y., Haas R.L. (2020). Time Trends and Prognostic Factors for Overall Survival in Myxoid Liposarcomas: A Population-Based Study. Sarcoma.

